# Stillbirths and quality of care during labour at the low resource referral hospital of Zanzibar: a case-control study

**DOI:** 10.1186/s12884-016-1142-2

**Published:** 2016-11-10

**Authors:** Nanna Maaløe, Natasha Housseine, Ib Christian Bygbjerg, Tarek Meguid, Rashid Saleh Khamis, Ali Gharib Mohamed, Birgitte Bruun Nielsen, Jos van Roosmalen

**Affiliations:** 1 Global Health Section, Department of Public Health, University of Copenhagen, Øster Farimagsgade 5, Building 9, 1353 Copenhagen K, Denmark; 2 Mnazi Mmoja Hospital, Zanzibar, Tanzania; 3 Julius Center for Health Sciences and Primary Care, University Medical Center Utrecht, Utrecht, The Netherlands; 4 School of Health & Medical Sciences, State University of Zanzibar, P.O.Box:146, Zanzibar, Tanzania; 5 Department of Obstetrics, Rigshospitalet, Copenhagen University Hospital, Blegdamsvej 9, 2100 Copenhagen Ø, Denmark; 6 Athena Institute, VU University of Amsterdam, De Boelelaan 1105, 1081 HV Amsterdam, The Netherlands

**Keywords:** Tanzania, Low resource, Stillbirths, Labour, Quality of care, PartoMa, Caesarean section, Severe hypertensive disorders, Oxytocin, Criterion-based audit, Case-control study, Guidelines, Partograph

## Abstract

**Background:**

To study determinants of stillbirths as indicators of quality of care during labour in an East African low resource referral hospital.

**Methods:**

A criterion-based unmatched unblinded case-control study of singleton stillbirths with birthweight ≥2000 g (*n* = 139), compared to controls with birthweight ≥2000 g and Apgar score ≥7 (*n* = 249).

**Results:**

The overall facility-based stillbirth rate was 59 per 1000 total births, of which 25 % was not reported in the hospital’s registers. The majority of singletons had birthweight ≥2000 g (*n* = 139; 79 %), and foetal heart rate was present on admission in 72 (52 %) of these (intra-hospital stillbirths). Overall, poor quality of care during labour was the prevailing determinant of 71 (99 %) intra-hospital stillbirths, and median time from last foetal heart assessment till diagnosis of foetal death or delivery was 210 min. (interquartile range: 75–315 min.). Of intra-hospital stillbirths, 26 (36 %) received oxytocin augmentation (23 % among controls; odds ratio (OR) 1.86, 95 % confidential interval (CI) 1.06–3.27); 15 (58 %) on doubtful indication where either labour progress was normal or less dangerous interventions could have been effective, e.g. rupture of membranes. Substandard management of prolonged labour frequently led to unnecessary caesarean sections. The caesarean section rate among all stillbirths was 26 % (11 % among controls; OR 2.94, 95 % CI 1.68–5.14), and vacuum extraction was hardly ever done. Of women experiencing stillbirth, 27 (19 %) had severe hypertensive disorders (4 % among controls; OR 5.76, 95 % CI 2.70–12.31), but 18 (67 %) of these did not receive antihypertensives. An additional 33 (24 %) did not have blood pressure recorded during active labour. When compared to controls, stillbirths were characterized by longer admissions during labour. However, substandard care was prevalent in both cases and controls and caused potential risks for the entire population. Notably, women with foetal death on admission were in the biggest danger of neglect.

**Conclusions:**

Intrapartum management of women experiencing stillbirth was a simple yet strong indicator of quality of care. Substandard care led to perinatal as well as maternal risks, which furthermore were related to unnecessary complex, time consuming, and costly interventions. Improvement of obstetric care is warranted to end preventable birth-related deaths and disabilities.

**Trial registration:**

This is the baseline analysis of the PartoMa trial, which is registered on ClinicalTrials.org (NCT02318420, 4th November 2014).

**Electronic supplementary material:**

The online version of this article (doi:10.1186/s12884-016-1142-2) contains supplementary material, which is available to authorized users.

## Background

More than a quarter of a million women and 2.7 million newborn babies lose their lives during pregnancy and childbirth annually [1, 2]. Though often invisible in global estimates, an additional 2.6 million stillbirths add profoundly to the tragedy, of which half are estimated to occur during labour [3]. In all three groups, the vast majority of deaths are caused by largely avoidable obstetric complications with the highest risk at the time of birth [1–3]. Many more women continue to suffer from birth related injuries, infections, and disabilities, and an estimated one million survivors of birth asphyxia may end up with cerebral palsy, learning difficulties, or other disabilities [4, 5]. The world’s highest burden of maternal and perinatal deaths and other birth-related complications remains in sub-Saharan Africa and Asia [1–3].

In the Millenium Development Goals’ era, the global strategy mainly aimed at skilled birth attendance, which resulted in campaigns for women to deliver in health facilities. The increasing proportion of facility births, however, has not been matched with improvements in the quality of intra-facility labour care [6, 7]. Notably, reports from referral hospitals in sub-Saharan Africa suggest ample room for improvement even at the tertiary level of the health care sector [8–11]. Importantly, these are the facilities where most of the countries’ future health care workers are trained, and if quality of care was improved, they could possibly be a lever for achieving nationwide health care improvements. Hence, in-depth insight into contextual challenges in delivering intrapartum quality of care is vital.

This paper is part of a baseline study for the PartoMa project, which aims at improving labour outcome for women and their offspring at the referral hospital of Zanzibar, Mnazi Mmoja Hospital. The project focuses on understanding direct and underlying determinants of substandard quality of care as well as strengthening monitoring and decision-making during labour [12]. We here present a case-control study of intrapartum management when the outcome was stillbirth. Although intrapartum stillbirths are considered a sensitive indicator of quality of care at the time of birth, there are few such studies from low income settings [3, 8, 11, 13, 14].

## Methods

### Setting

The Zanzibar archipelago, a semiautonomous part of Tanzania, struggles with poverty and a resource constraint health system. Half of the 1.3 million Zanzibarians live below the poverty line [15]. In 2011, the maternal mortality ratio was reported at 287 deaths per 100 000 live births, of which the majority occurs during or shortly after childbirth [16]. Though little is known about perinatal mortality, estimates from 2010 suggest a rate of 50 perinatal deaths per 1000 total births, as opposed to 36 per 1000 in mainland Tanzania [17].

The governmental Mnazi Mmoja Hospital is the only tertiary care facility on the archipelago, and at the time of data collection, the only hospital on the biggest island providing comprehensive obstetric and neonatal care around the clock. In 2014, 13 291 women delivered in the hospital, corresponding with an average of 36 deliveries daily. Of these, 16 % were caesarean sections (CSs), and 44 maternal deaths were counted. According to the official registers, 41 babies were stillborn in 2014 per 1000 total births, and 17 neonatal deaths occurred up to discharge per 1000 live births.

The hospital plays a leading role in clinical training of future Tanzanian health care providers. Yearly, approximately 60 intern doctors do their initial clinical rotations at the department, and more than 200 Tanzanian nursing and medical students are trained.

Intrapartum care is located in two rooms only. In the labour room’s 18 beds, women are assisted during the first stage of labour. Postpartum women needing extra surveillance stay in this room as well. When women reach the second stage of labour, they walk 15 m to the delivery room with only three beds. There is one, occasionally two, theatres available for obstetric surgery. For more than a decade, it has been the aim to apply the World Health Organization’s (WHOs) composite partograph for all women in labour, which is a graphic monitoring sheet including foetal heart rate (FHR), labour progress, and maternal vital signs during latent and active phase of labour [18]. A treatment and observation sheet for severe hypertensive disorders has been available since June 2014 and includes evidence-based guidelines on anticonvulsant and antihypertensive treatment [19].

After uncomplicated delivery, the woman and baby are usually observed in the labour room for two hours before referral to the postnatal ward for another four hours where no routine observations are done. Babies with birth asphyxia, with birthweights <1500 gram (g), or delivered by CS are admitted to the Neonatal Intensive Care Unit, which is adjacent to the delivery room.

At day time, there are on average three nurse-midwives and two registrar doctors on duty in the labour and delivery rooms as well as a fluctuating number of intern doctors. During evenings and nights, there are two or three nurse-midwives on duty for all obstetric patients and one registrar doctor assisted by two interns are in charge of the entire department, which also includes an average of 30 gynaecological patients. A second doctor is on call from home. At the department, there are two specialist obstetricians, of which one is member of the study team (TM).

### Study population

All stillbirths defined as late foetal deaths ≥1000 g [20] were identified between 1st October 2014 and 31st January 2015 in the admission, delivery, and theatre registers, and their case files were searched for. In addition, to give the best possible estimate of the actual hospital-based stillbirth rate, non-registered stillbirths were sought by systematically going through all case files from the four months. Due to a breakdown in the hospital’s storage system of case files prior to October 2015, four months was the maximum feasible period for the study. The control group was identified in the same registers and from the same time period. The first control included in October 2014 was selected by throwing dices and afterwards every tenth delivery was identified, resulting in a case control ratio of approximately 1:4. These tasks were conducted by three research assistants (NH, RSK, and AGM), who had weekly meetings with TM or NM to assure quality of files retrieval.

**Fig. 1:**
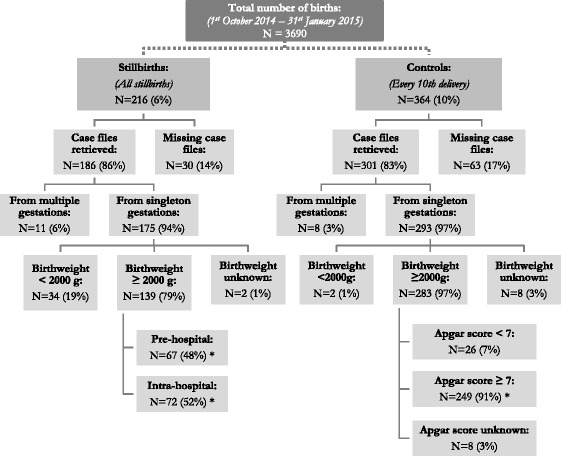
Sampling of case files. Facility-based stillbirth rate was 59 per 1000 total births. Stillbirths: All late foetal deaths with birthweight ≥1000 g. Pre-hospital stillbirths: No documented positive foetal heart rate on admission. Intra-hospital stillbirths: Documented positive foetal heart rate on admission. *Groups compared by the case-control study

Afterwards, all case files were checked individually by NM to determine whether the inclusion criteria were met for in-depth criterion-based audit (Fig. 1).

All singleton stillbirths were included for the audit and divided into three groups: birthweight 1000–1999 g (very preterm stillbirths) [21]; birthweight ≥2000 g without positive FHR on admission (pre-hospital stillbirths), and birthweight ≥2000 g with positive FHR on admission (intra-hospital stillbirths). Hence, pre-hospital stillbirths included not only cases in which the stillbirth diagnosis was made on admission, but also cases where there was no documentation of FHR (neither present nor absent) during the admission. While deliveries from multiple gestations were included in the overall estimation of the stillbirth rate, they were not included in the further audit process as their case files were often too ambiguous; e.g. frequently only one FHR was registered throughout labour. The 2000 g cut-off was decided as this reflects gestational age of 32–34 weeks, where lung maturity no longer plays a major role in survival, and the newborn is less dependent on dexamethasone treatment prior to delivery as well as advanced intensive neonatal care [22]. Data on the very preterm stillbirths are only presented in Additional file 1.

Inclusion criteria for controls were singletons with birthweight ≥2000 g and Apgar score ≥7; hence a group with immediate good neonatal outcome. It varied whether one, five or 10 min (min.) Apgar scores were recorded. It was therefore decided to use the latest. The case-control study was unblinded.

### Criteria of realistic best quality of care

Criteria reflecting locally best possible labour management with the available resources were formulated and agreed upon. This developmental process was conducted by a participatory approach including both local skilled birth attendants and seven external specialists in midwifery and obstetrics. It was decided that the standards should include routine labour care as well as management of frequent complications of labour, related to FHR and foetal distress, labour progression by dilatation and descent, and maternal vital signs with specific focus on severe hypertensive disorders and fever. Selected criteria were adapted from the Active management of labour package, modified by WHO, and supplemented with other evidence-based guidelines [23–31]. Frequency of routine assessments was reduced to reflect local reality. For example, FHR assessments every 30 min. for all women in active labour were kept as optimal practice, but assessments within intervals of <90 min. were applied as an acceptable audit criterium.

In addition, information was collected on background and admission characteristics as well as outcome parameters. Finally, if information was available, time from last FHR assessment till delivery or diagnosis of intrauterine foetal death was calculated, as well as the admission to delivery interval.

### Data extraction and analysis

Data was extracted into a structured entry form based on the pre-selected audit criteria, using EPI INFO 7 software (Centres for Disease Control and Prevention, Atlanta, GA, USA). Differences were analysed by logistic regression in SAS Enterprise Guide 6.1 (SAS Institute, Inc., Cary, NC). *P*-values <0.05 were considered statistically significant.

## Results

During the four months studied, 216 stillbirths occurred out of 3690 total births. This corresponds to an overall hospital-based stillbirth rate of 59 per 1000 total births, of which 53/216 (25 %) were not reported in the hospital’s official registers. Case files of 186/216 (86 %) stillbirths and 301/364 (83 %) controls could be retrieved, of which 175/186 (94 %) and 293/301 (97 %) were from singleton gestations. Of the singleton stillbirths, 139/175 (79 %) had a birthweight ≥2000 g and were included in the case-control study. Of these, 72/139 (52 %) had a positive foetal heart rate on admission (intra-hospital stillbirths), with only one having a congenital abnormality that may have been incompatible with life. Of the 67 pre-hospital stillbirths, 20/67 (30 %) had no documentation of FHR readings during the admission. Classification in ‘fresh’ and ‘macerated’ stillbirths was not recorded in 77/139 (55 %) stillbirths, and it was therefore not useful for determining the rate of intrapartum deaths. Of controls, 249 met the inclusion criteria (Fig. 1).

### Background characteristics

Among intra-hospital stillbirths, 39/72 (54 %) were nulliparous versus 14/64 (22 %) pre-hospital stillbirths (odds ratio [OR] 4.22, 95 % confidence interval [CI] 1.99–8.96) and 105/239 (44 %) controls (OR 1.51, 95 % CI 0.89–2.56). All women, except one, had attended antenatal care at least once. Of women experiencing stillbirth, 69/119 (58 %) had attended at least four visits, versus 103/214 (48 %) controls (OR 1.49, 95 % CI 0.95–2.34). Cases with missing information are excluded from these comparisons (Table 1).

**Table 1:** Characteristics of delivering women

	Case-control study BW ≥2000 g		
	Cases Pre-hosp. Stillbirths	Cases Intra-hosp. Stillbirths	Controls *Apgar 7*–*10*
	*N* (%)		
*Of all women in the study:*	*(n* = *67)*	*(n* = *72)*	*(n* = *249)*
Age			
<20 years	2 (3.0 %)	7 (9.7 %)	26 (10.4 %)
20–29 years	26 (38.8 %)	35 (48.6 %)	122 (49.0 %)
30–39 years	27 (40.3 %)	28 (38.9 %)	83 (33.3 %)
≥40 years	8 (11.9 %)	2 (2.8 %)	15 (6.0 %)
Information missing	4 (6.0 %)	0 (0.0 %)	3 (1.2 %)
Parity on admission			
Para 0^a^	14 (20.9 %)	39 (54.2 %)	105 (42.2 %)
Para 1–4	33 (49.3 %)	23 (31.9 %)	99 (39.8 %)
Para ≥ 5	17 (25.4 %)	10 (13.9 %)	35 (14.1 %)
Information missing	3 (4.5 %)	0 (0.0 %)	10 (4.0 %)
Antenatal care			
≥4 visits	31 (46.3 %)	38 (52.8 %)	103 (41.4 %)
1–3 visits	23 (34.3 %)	26 (36.1 %)	111 (44.6 %)
Not attended	0 (0.0 %)	1 (1.4 %)	0 (0.0 %)
Information missing	13 (19.4 %)	7 (9.7 %)	35 (14.1 %)
HIV			
Negative	54 (80.6 %)	62 (86.1 %)	211 (84.7 %)
Positive	0 (0.0 %)	2 (2.8 %)	0 (0.0 %)
Information missing	13 (19.4 %)	8 (11.1 %)	38 (15.3 %)
Gestational age			
No information on LMP/gestation weeks	46 (68.7 %)	49 (68.1 %)	181 (72.7 %)
Previous obstetric history			
*Of multiparous women:*	*(n* = *50)*	*(n* = *33)*	*(n* = *134)*
Previous death of child/children^b,^ ^c^	18 (36.0 %)	12 (36.4 %)	30 (22.4 %)
1 previous CS	7 (14.0 %)	8 (24.2 %)	8 (6.0 %)
≥2 previous CSs	2 (4.0 %)	2 (6.1 %)	10 (7.5 %)

*BW* birthweight, *CI* confidence interval, *CS* caesarean section, *LMP* last menstrual period, *OR* odds ratio

^a^Difference between pre- and intra-hospital stillbirths: OR 4.22, 95 % CI 1.99–8.96

^b^Documentation was insufficient to clearly distinguish perinatal deaths from deaths later in life

^c^Difference between stillbirths and controls: OR 1.96, 95 % CI 1.07–3.59

Of multiparous women experiencing stillbirth, 30/83 (36 %) had also previously experienced the loss of one or more children, compared to 30/134 (22 %) controls (OR 1.96, 95 % CI 1.07–3.59). A history of previous CS occurred in 19/83 (23 %) compared to 18/134 (13 %) controls (OR 1.91, 95 % CI 0.94–3.90; Table 1).

Of stillbirths, 35/135 (26 %) were delivered by CS, compared to 26/244 (11 %) controls (OR 2.94, 95 % CI 1.68–5.14). While 10/35 (26 %) of the CSs resulting in stillbirth were performed in the second stage of labour, this was never the case for controls. Only one baby was delivered by vacuum extraction. Of intra-hospital stillbirths delivered by CS, 10/20 (50 %) had FHR documented at the time of deciding on CS. In five of these ten women, FHR were absent. Foetal distress was recorded as an indication for CS in only one case. Indications for CSs resulting in stillbirth were: 21/35 (60 %) for prolonged labour, of which one was also due to foetal distress; 8/35 (23 %) due to antepartum haemorrhage, of which three had severe pre-eclampsia; an additional 2/35 (6 %) solely due to severe pre-eclampsia; 2/35 (6 %) due to cord prolapse; and 2/35 (6 %) due to ≥2 previous CSs. Concerning the prolonged labour group, 1/21 (5 %) had signs of obstructed labour on admission and another 6/21 (29 %) developed uterine rupture; half had signs of rupture on admission, of which two were referrals, and three ruptures occurred at the study site. Four of the women with uterine rupture had a history of previous CS (Table 2).

**Table 2:** Mode of delivery, maternal outcome, and appearance of stillborn babies

	Case-control study BW ≥2000 g		
	Cases Pre-hosp. Stillbirths	Cases Intra-hosp. Stillbirths	Controls *Apgar 7*–*10*
	*N* (%)		
Mode of delivery			
*Of all women in the study:*	*(n* = *67)*	*(n* = *72)*	*(n* = *249)*
Spontaneous vaginal	45 (67.2 %)	46 (63.9 %)	213 (85.5 %)
Vaginal breech	3 (4.5 %)	5 (6.9 %)	5 (2.0 %)
Vacuum extraction	1 (1.5 %)	0 (0.0 %)	0 (0.0 %)
Caesarean section^a,^ ^b^	15 (22.4 %)	20 (27.8 %)	26 (10.4 %)
Mode of delivery unknown	3 (4.5 %)	1 (1.4 %)	5 (2.0 %)
Maternal outcome			
*Of all women in the study:*	*(n* = *67)*	*(n* = *72)*	*(n* = *249)*
Maternal deaths	2 (3.0 %)	1 (1.4 %)	0 (0.0 %)
Post partum haemorrhage^c^	7 (10.4 %)	10 (13.9 %)	14 (5.6 %)
Episiotomy/spontaneous tears^d,^ ^e^	6 (9.0 %)	19 (26.4 %)	79 (31.7 %)
*Of vaginal deliveries:*	*(n* = *49)*	*(n* = *51)*	*(n* = *218)*
Prolonged admission, ≥1 day^f^	9 (18.4 %)	0 (0.0 %)	3 (1.4 %)
*Of caesarean sections:*	*(n* = *15)*	*(n* = *20)*	*(n* = *26)*
Prolonged admission, ≥5 days	1 (6.7 %)	3 (15.0 %)	2 (7.7 %)
‘Fresh’ versus ‘macerated’ stillbirths			
*Of all women in the study:*	*(n* = *67)*	*(n* = *72)*	*(n* = *249)*
Classification not recorded	36 (53.7 %)	41 (56.9 %)	NA

*BW* birthweight, *CI* confidence interval, *NA* not applicable; OR, odds ratio

^a^Overall, 9/35 (26 %) of the caesarean sections with stillbirth were done prior to active labour, and 10/35 (29 %) in second stage. Among controls, this was the case for 13/26 (50 %) and 0/26 (0 %), respectively

^b^Difference between stillbirths and controls: OR 2.94, 95 % CI 1.68–5.14

^c^Difference between stillbirths and controls: OR 2.34, 95 % CI 1.12–4.90

^d^Information was insufficient to distinguish between spontaneous vaginal tears and episiotomies

^e^Difference between pre-hospital stillbirths and controls: OR 0.21, 95 % CI 0.09–0.51

^f^Difference between pre-hospital stillbirths and controls: OR 16.13, 95 % CI 4.18–62.17

There were three maternal deaths in the study population. They were all associated with stillbirth and suffered from severe delays in intrahospital surveillance and management (Additional file 2).

### Admission and partograph use

Women experiencing intra-hospital stillbirths were admitted particularly early; 42/71 (59 %) before the active phase of labour, compared to 68/246 (28 %) controls (OR 3.79, 95 % CI 2.19–6.57). Their median time from admission to delivery was 11 h and 36 min. (interquartile range (IQR): 5 h and 42 min. to 21 h and 55 min.). Rates of referrals were similar among pre- and intra-hospital stillbirths. The referral rate among stillbirths (21/139; 15 %) was higher than the rate among controls (12/249 (5 %); OR 3.52, 95 % CI 1.67–7.39; Table 3).

**Table 3:** Admission and partograph use

	Case-control study BW ≥2000 g		
	Cases Pre-hosp. stillbirths	Cases Intra-hosp. Stillbirths	Controls *Apgar 7*–*10*
	*N* (%)		
Progress on admission and referrals			
*Of all women in the study:*	*(n* = *67)*	*(n* = *72)*	*(n* = *249)*
Before labour pain^a^	5 (7.5 %)	2 (2.8 %)	12 (4.8 %)
Latent phase of labour^a,^ ^b^	18 (26.9 %)	40 (55.6 %)	56 (22.5 %)
First stage of labour	23 (34.3 %)	29 (40.3 %)	153 (61.4 %)
Second stage of labour	15 (22.4 %)	0 (0.0 %)	25 (10.0 %)
Stage of labour on admission unknown	6 (9.0 %)	1 (1.4 %)	3 (1.2 %)
Referral from smaller health centre^c^	10 (14.9 %)	11 (15.3 %)	12 (4.8 %)
Partograph use			
*Of women in first stage of labour:*	*(n* = *39)*	*(n* = *69)*	*(n* = *207)*
The partograph at least partially applied^d^	27 (69.2 %)	66 (95.7 %)	183 (88.0 %)
*Of women with the partograph applied:*	*(n* = *27)*	*(n* = *66)*	*(n* = *183)*
First cervical dilatation in active labour plotted correctly on the alert line	18 (66.7 %)	53 (80.3 %)	166 (90.7 %)

*BW* birthweight, *CI* confidence interval, *OR* odds ratio

^a^Difference in women admitted before active labour between intra-hospital stillbirths and controls: OR 3.79, 95 % CI 2.19–6.57

^b^Cervical dilatation <4 cm

^c^Difference between intra-hospital stillbirths and controls: OR 3.52, 95 % CI 1.67–7.39

^d^Difference between pre-hospital stillbirths and both intra-hospital stillbirths and controls: OR 9.78, 95 % CI 2.56–37.42, and OR 3.39, 95 % CI 1.52–7.56, respectively

**Fig. 2:**
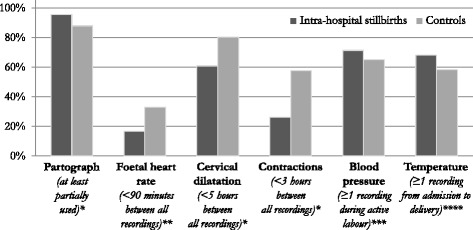
Proportion of labouring women reaching each of six criteria for minimal acceptable routine surveillance during labour. Significant differences were found in FHR (OR 0.41, 95 % CI 0.21–0.81), cervical dilatation (OR 0.37, 95 % CI 0.21–0.68), and contractions (OR 0.26, 95 % CI 0.14–0.47). Intra-hospital stillbirths: documented positive FHR on admission, birthweight ≥2000 g. Controls: Apgar score ≥7, birthweight ≥2000 g. * Of all women at the hospital during active first stage of labour (*n* = 69 and *n* = 207, respectively). ** Of women with at least one FHR reading (*n* = 72 and *n* = 204, respectively). *** Of women reaching active phase of labour (*n* = 70 and *n* = 235, respectively). **** Of all women in the study (*n* = 72 and *n* = 249, respectively). FHR, foetal heart rate; BP, blood pressure; Temp, temperature

Of women reaching active labour and admitted before second stage, significantly more in the pre-hospital stillbirth group did not have a partograph filled in, when compared to both intra-hospital stillbirths (OR 9.78, 95 % CI 2.56–37.42) and controls (OR 3.39, 95 % CI 1.52–7.56; Fig. 2). In all groups, 237/276 (86 %) women with a partograph applied had the first cervical dilatation appropriately plotted on the alert line (Table 3).

### Foetal heart rate (FHR)

In all intra-hospital stillbirths, FHR was reassuring on admission. However, in 60/72 (83 %) >90 min. elapsed between FHR assessments during active phase of labour, which was the case for 137/204 (67 %) controls (OR 2.45, 95 % CI 1.23–4.85; Fig. 2). Among 63 intra-hospital stillbirths, median time from last FHR recording till delivery or detected intrauterine foetal death was 3 h and 30 min. (IQR: 1 h and 15 min.–5 h and 15 min.), compared to 2 h and 0 min. in 176 controls (IQR: 1 h and 3 min.–3 h and 58 min.). For each one-hour increase in duration from last FHR assessment, the odds of stillbirth increased 20 % (OR 1.20; 95 % CI 1.08–1.34). In 58/72 (81 %) of the intra-hospital stillbirths, there was no documentation of foetal distress or foetal death prior to delivery (Table 4).

**Table 4:** Intrapartum surveillance of the foetus

	Case-control study BW ≥2000 g	
	Cases Intra-hosp. Stillbirths	Controls *Apgar 7*–*10*
	*N* (%)	
*Of women with at least one FHR reading:*	*(n* = *72)*	*(n* = *204)*
FHR in normal range on admission (110–160 beats per min.)	72 (100.0 %)	202 (99.0 %)
Foetal distress detected prior to delivery	15 (20.8 %)	0 (0.0 %)
<90 min. between any 2 recordings of FHR^a^	12 (16.7 %)	67 (32.8 %)
Median time from last FHR till delivery or detected IUFD (min.)^b,^ ^c^	210	120

*BW* birthweight, *CI* confidence interval, *FHR* foetal heart rate, *min.* minutes, *OR* odds ratio

^a^Difference between intra-hospital stillbirths and controls: OR 0.41, 95 % CI 0.21–0.81

^b^It was possible to calculate average time from last FHR till delivery in 63 (86 %) cases and 176 (86 %) controls. The interquartile ranges were 75–315 min. and 63–238 min., respectively

^c^For each one-hour increase in duration from last FHR assessment, the odds of stillbirth increased 20 % (OR 1.20; 95 % CI 1.08–1.34)

### Labour progress

The highest proportion of women admitted in the latent phase of labour with no cervical assessments recorded during active labour occurred among pre-hospital stillbirths: 14/23 (61 %) compared to 24/68 (35 %) controls (OR 2.85, 95 % CI 1.08–7.55; Table 5).

**Table 5:** Intrapartum surveillance of labour progress

	Case-control study BW ≥2000 g		
	Cases Pre-hosp. Stillbirths	Cases Intra-hosp. Stillbirths	Controls *Apgar 7*–*10*
	*N* (%)		
Surveillance in latent phase of labour			
*Of women admitted before active labour:*	*(n* = *23)*	*(n* = *42)*	*(n* = *68)*
Assessment of cervical dilatation during active labour^a,^ ^b^	9 (39.1 %)	37 (88.1 %)	44 (64.7 %)
Assessment of labour progression			
*Of women in first stage of active labour:*	*(n* = *39)*	*(n* = *69)*	*(n* = *207)*
<5 h. between any 2 recordings of cervical dilatation in active labour ^c^	39 (100.0 %)	42 (60.9 %)	167 (80.3 %)
<3 h. between any 2 recordings of uterine contractions^d^	33 (84.6 %)	18 (26.1 %)	120 (58.0 %)
Alert line crossed^e^	2 (5.1 %)	33 (47.8 %)	51 (24.5 %)
Action line crossed^f^	1 (2.6 %)	16 (23.2 %)	21 (10.1 %)

*BW* birthweight, *CI* confidence interval, *OR* odds ratio

^a^If a vaginal examination was done in latent phase ≤4 h prior to delivery, this was registered as acceptable

^b^Difference between pre-hospital stillbirths and controls: OR 0.35, 95 % CI 0.13–0.93

^c^Difference between intra-hospital stillbirths and controls: OR 0.37, 95 % CI 0.21–0.68

^d^Difference between intra-hospital stillbirths and controls: OR 0.26, 95 % CI 0.14–0.47

^e^Difference between intra-hospital stillbirths and controls: OR 2.80, 95 % CI 1.59–4.95

^f^Difference between intra-hospital stillbirths and controls: OR 2.67, 95 % CI 1.30–5.49

In 27/69 (39 %) women experiencing intra-hospital stillbirth, ≥5 h elapsed between any two vaginal examinations during active labour, compared to 40/207 (19 %) controls (OR 2.68, 95 % CI 1.48–4.86; Fig. 2). This resulted in delays in diagnosing poor labour progress, which was a common complication among intra-hospital stillbirths when compared to controls (Table 5). After crossing the alert line, in 18/33 (55 %) and 9/51 (18 %), respectively, ≥3 h elapsed before next vaginal examination (OR 5.60, 95 % CI 2.07–15.13). After crossing the action line, in 2/16 (13 %) intra-hospital stillbirths and 9/21 (43 %) controls, membranes were still intact, and in an additional 3/16 (19 %) and 5/21 (24 %), there was no information regarding membranes. Moreover, severe delays in surveillance were found after crossing the action line.

**Fig. 3:**
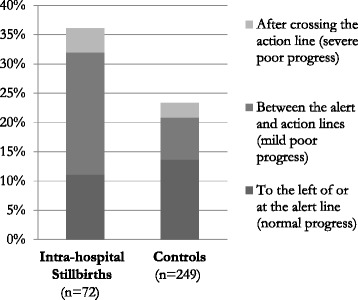
Initiation of oxytocin for labour augmentation, according to labour progress. The difference in overall use of oxytocin for labour augmentation between intra-hospital stillbirths and controls was significant with the stillbirth cases receiving the treatment more often (OR 1.86, 95 % CI 1.06–3.27). Intra-hospital stillbirths: Documented positive foetal heart rate on admission, birthweight ≥2000 g. Controls: Apgar score ≥7, birthweight ≥2000 g

Oxytocin for labour augmentation was administered in 26/72 (36 %) of intra-hospital stillbirths, compared to 58/249 (23 %) controls (OR 1.86, 95 % CI 1.06–3.27). However, in 8/26 (31 %) of those, there was no indication for augmentation, and in an additional 7/26 (27 %) the infusion was started between the alert and action line with the membranes still intact. Likewise, 34/58 (59 %) controls had the infusion started before crossing the alert line (Fig. 3). In none of the cases, information on oxytocin titration and maintenance dose was documented, and FHR and contractions were never assessed half hourly after starting the infusion. Preceeding 13/35 (37 %) of CSs resulting in stillbirth, oxytocin was started for labour augmentation. This was also the case in four of six uterine ruptures, of which three had a history of one previous CS.

### Maternal vital signs

Prevalence of severe hypertensive disorders was significantly higher among women experiencing stillbirth (27/139, 19 %) than in controls (10/249, 4 %; OR 5.76, 95 % CI 2.70–12.31). An additional 33/130 (25 %) and 81/235 (35 %) of women reaching active phase of labour, had no recordings of blood pressure during active labour (OR 0.65, 95 % CI 0.40–1.04; Fig. 2). Overall, 13/27 (48 %) of all stillbirth cases with severe hypertension had significant proteinuria (≥2+ on urine dipstick). However, urine analysis was not recorded in an additional 6/27 (22 %) cases, and information about clinical symptoms were too sparse to analyse for signs of organ failure. In 18 (67 %) of all 27 stillbirth cases with severe hypertension, there were no recordings of relevant antihypertensive treatment. Of the severe pre-eclampsia cases, 4/13 (31 %) had no documentation of having received magnesium sulphate.

In 43/139 (31 %) stillbirths and 104/249 (42 %) controls, there was no maternal temperature recording from admission till delivery (OR 0.63, 95 % CI 0.40–0.97; Fig. 2). Intrapartum fever or infection were rare diagnoses with five stillbirths related to infection and none among controls.

## Discussion

The overall facility-based stillbirth rate was 59 per 1000 total births. Approximately 80 % of the singleton stillbirths had a birthweight ≥2000 g. In half of these, the FHR was still present after admission to hospital. In all groups, major challenges were identified in intrapartum surveillance, timely decision-making, and documentation. This resulted in stillbirths as well as unacceptable maternal and neonatal risks for all women and babies. The findings are largely in line with the limited number of other stillbirth studies from sub-Saharan Africa [3, 11, 13, 14]. Our study provides a more in-depth assessment of intrapartum care, which may contribute to effectively target interventions to reduce risks through improved quality of care (Table 6).

**Table 6:** Seven target areas for improving intrapartum quality of care at the study site

1. Strengthened risk assessment on admission, with particular focus on foetal heart rate, blood pressure, temperature, and previous obstetric history.
2. Improved routine surveillance during latent and active phase of labour, regarding all key parameters (foetal heart rate, dilatation of cervix and descent, contractions, maternal vital signs, and urinary output).
3. Increased prioritization of women with already diagnosed intrauterine foetal death for routine assessments during labour.
4. Timely prevention and management of prolonged labour, with focus on alternative and less harmful interventions than oxytocin infusion for labour augmentation (e.g. artificial rupture of membranes and emptying of bladder), and more restrictive dosages and improved surveillance when oxytocin is administered.
5. Reduction of caesarean sections after intrauterine foetal death, by improved management of prolonged labour, and enforcement of vacuum extraction and craniotomy use.
6. Improved management of severe hypertensive disorders, with particular focus on antihypertensive treatment.
7. Better intrapartum documentation as well as record keeping.

### Causes of stillbirths

As suggested in other studies, the high number of intra-hospital stillbirths appeared primarily to be a sensitive indicator of substandard quality of care [3, 32, 33]. For instance, primigravid women suffered an increased risk of intra-hospital stillbirths, which may be associated with their admission earlier in labour, and longer and more complicated labour duration [34]. In addition, increased vulnerability and need for quality care among all women experiencing intra-hospital stillbirth is emphasized by the association with severe hypertension and prior loss of child.

While staff often referred to late referral of women as a major cause to adverse labour outcome, 85 % of women experiencing intra-hospital stillbirth directly sought care at the study site, and 58 % were admitted before active labour.

As found in other East African studies [8, 11, 35], not only was correct management often delayed, unnecessary and even harmful management was sometimes initiated. The presence of under- as well as over-treatment became particularly apparent when reviewing cases with poor progress of labour, where CSs often were found unnecessary. Yet, as only 4 % of foetuses had a documented positive FHR when deciding to perform CS, it is doubtful how big an impact e.g. possible delays in the decision-to-delivery interval for CS had on causing stillbirths; the unnecessary CSs may rather have caused avoidable maternal risks. In contrast, the under-use of vacuum extraction may be seen as an indicator of poor FHR monitoring, leading to undisclosed foetal distress in the second stage of labour.

Likewise, there appeared to be a dangerous over-use of oxytocin for labour augmentation. This is similar to studies from Bangladesh and Pakistan, where misuse of oxytocin was associated with stillbirth and birth asphyxia [36, 37]. Notably, the Pakistani study draws attention to the danger of insufficiently trained healthcare workers administrating this highly potent drug [36].

Similar to intrapartum care, antenatal visits did not appear consistent with effective antenatal surveillance and treatment, and as in other studies this appeared to be a central determinant of both pre- and intra-hospital stillbirths [33]. For instance, while nearly all women attended antenatal care at least once, a severe hypertension prevalence of 19 % among stillbirths suggests missed opportunities [38]. Furthermore, the fact that less than half of the study population had attended four antenatal care visits indicate a lost chance for continuity in care [39].

### Maternal risks

Substandard quality of risk assessment on admission as well as poor intrapartum surveillance and decision-making were associated with profound maternal risks and appeared to be major determinants of the death of three women (Additional file 2). Women with foetal death on admission were the most neglected. While they were in particular high intrapartum risk due to the often underlying morbidity and further at increased postpartum risk of e.g. obstetric fistula, labour progress and vital signs were often undocumented throughout active labour [40, 41].

CS is generally not indicated when there is foetal death [42]. However, a high proportion of CSs were done on doubtful indications, and many were related to insufficient management of prolonged labour. Thus, 26 % CSs among stillbirths is unacceptably high; in particular as the vast majority had either foetal death diagnosed or did not have FHR recorded prior to surgery. Except for an even higher rate found at three hospitals in Mozambique [11], this is markedly higher than other studies from low- and middle-income countries [33]. A high proportion of CSs were done in the second stage of labour without an attempt of operative or destructive vaginal delivery. While short- and long-term maternal risks of suboptimally treated prolonged labour and unnecessary CSs are widely established [34, 43, 44], lack of transparency as to when to perform CS is found in other African studies too [11, 45, 46].

Six women suffered from uterine rupture. When considering the low level of surveillance in 14 % of stillbirth cases with one or more previous CSs, and the misuse of oxytocin, many more appeared at risk of rupture. Furthermore, while foetal bradycardia is an early sign of impending rupture [47], substandard FHR assessments made it less useful in timely detection.

Of the 19 % with severe hypertensive disorders experiencing stillbirth, more than half had severe pre-eclampsia or eclampsia. A Nigerian study found a similar prevalence and comparable insufficient antenatal and intrapartum surveillance and treatment of these dangerous conditions [48]. Recent data from well-resource settings emphasize suboptimally treated severe hypertension as an important contributor to maternal mortality [38]; in our study, 67 % of women with severe hypertension did not receive any antihypertensive treatment.

### Clinical implications

At this East African referral hospital, facility births were frequently not accompanied by skilled intrapartum attendance. While widespread insufficiency in quality of routine and emergency labour care may partly be caused by massive structural constraints, suboptimal care often resulted in more risk associated, complicated, and resource draining interventions. Some of the revealed deficiencies may be addressed even without high costs in manpower and other resources, and the main risks and determinants are crucial in effectively designing low cost interventions (Table 6).

For many years, effectiveness of using the WHO partograph has been questioned [49, 50]. Yet, when analysing quality of intrapartum care at low resource facilities, partograph use for timely surveillance and decision-making appears central in ending preventable complications [11, 14, 18, 35]. In 86 % of all cases where the partograph was applied, first cervical dilatation in active phase of labour was plotted correctly on the alert line, and knowledge on accurate recording did not appear to be a major challenge. However, similar to other studies, WHO’s recommendations for frequency of recordings were not followed and also did not seem achievable with the resources available [35, 51, 52]. In the present study, even though the majority of intrapartum decision-making did not seem influenced by partograph use or evidence-based guidelines, it would be premature to conclude ineffectiveness of the WHO partograph. However, for the partograph to assist in surveillance and management, it must be coupled to a locally achievable and relevant labour management protocol. Although often not prioritised in evaluations of partograph use, this has previously proven effective [14, 18]. For instance, when considering the low resources at the study site, it seems unrealistic to assure close monitoring and titration of oxytocin augmentation if more than a few women are treated simultaneously [53].

This study identified 25 % underreporting of stillbirths in the official hospital registers, and even though a systematical surge was conducted through all piles of case files, a considerable number of files remained missing (Fig. 1). Initially, it was the intention also to include early neonatal deaths in the study. However, data collection revealed frequent default record keeping between the obstetric and neonatal units as well as substantial underreporting of very early neonatal deaths in all registers, which resulted in reluctance to include them. Furthermore, missing documentation in medical records – or “blanks” – was a frequent finding, which is likely to have affected patient care and labour outcomes. Incomplete health information systems are notoriously linked with poor health outcomes [3, 54]. It is warranted that the underlying factors for these “blanks” in medical recording are evaluated, and that quality of documentation and record keeping as well as use of the data are improved.

### Strengths and limitations

The present pragmatic study was found suitable as a structured, simple, and low-cost method to identify central challenges in intrapartum care at this real-world setting with limited information available. Classification in pre- and intra-hospital stillbirths was a useful, more achievable, and simple alternative to ‘fresh’ versus ‘macerated’ stillbirths, which, as in a study from Ghana, was found unreliable [55]. Moreover, intra-hospital stillbirths may be seen as an even stronger indicator of intra-hospital quality of care than ‘fresh’ stillbirths. However, in 30 % of pre-hospital stillbirths there was no FHR documentation on or after admission, which may potentially hide an even higher proportion of intra-hospital stillbirths.

Selected audit criteria were unambiguously applicable. Yet, though intensive efforts were made for adapting international evidence-based guidelines to reach local reality, some criteria, such as <90 min. between FHR recordings, might be too optimistic as a sensitive audit standard for detecting quality improvements at this setting.

A central limitation to the study is that a criterion-based audit does not allow exploration of underlying determinants of substandard care, such as structural needs for supplies, space, and knowledge/skills among staff. Another limitation is the varying quality of data, which might bias results; there may be a tendency of staff to forget reporting given care or to under-report mismanagement. Participant observations during the study period also identified the issues presented in the current paper, and qualitative analysis opened up to a complex tangle of both structural and process related underlying challenges influencing health providers’ ability to deliver acceptable quality of care.

## Conclusion

Stillbirths are both a devastating burden of avoidable lost lives in itself and a strong and easy to assess indicator of quality of antenatal and intrapartum care. Substandard care led to substantial maternal and perinatal risks, which furthermore were related to resource draining interventions that were not always necessary. Furthermore, 25 % underreporting of stillbirths in hospital registers indicates a poor health information system. These findings are largely in line with other reports from sub-Saharan Africa, and improvement of intra-hospital obstetric knowledge, care, and documentation is central to end preventable birth-related deaths and disabilities. Considering referral hospitals’ major teaching tasks for future health providers, it is warranted to address the tertiary level in order to achieve quality improvement of the entire health care sector.

## Additional files

Additional file 1: Very preterm stillbirths. Criterion-based audit data on very preterm stillbirths (birthweight <2000 g). (DOCX 49 kb)
Additional file 2: Maternal deaths. Intra-hospital management preceding the three maternal deaths within the study population. (DOCX 39 kb)

## Abbreviations

CI: Confidence interval
CS: Caesarean section
FHR: Foetal heart rate
g: Grams
IQR: Interquartile range
min.: Minutes
OR: Odds ratio
WHO: World Health Organization
