# Supplementary material for: Stillbirths and quality of care during labour at the low resource referral hospital of Zanzibar: a case-control study
Source: BMC Pregnancy Childbirth. 2016 Nov 10;16:351. doi: 10.1186/s12884-016-1142-2 (PMC5103376; doi:10.1186/s12884-016-1142-2)
Supplement: Additional file 1: — Very preterm stillbirths. Criterion-based audit data on very preterm stillbirths (birthweight <2000 g). (DOCX 49 kb) [file 12884_2016_1142_MOESM1_ESM.docx]

**Additional file 1: Data on very preterm stillbirths (birthweight <2000 grams)**

**Table S1** Background characteristics for very preterm stillbirths

|  | **Very preterm stillbirths** *BW 1000-1999 g* |
| --- | --- |
|  | N (%) |
| *Of all women in the study:* | *(n=34)* |
| **Age** |  |
| <20 years | 0 (0.0%) |
| 20-29 years | 15 (44.1%) |
| 30-39 years | 14 (41.2%) |
| ≥40 years | 0 (0.0%) |
| Information missing | 5 (14.7%) |
| **Parity on admission** |  |
| Para 0 | 10 (29.4%) |
| Para 1-4 | 18 (52.9%) |
| Para ≥5 | 6 (17.6%) |
| Information missing | 0 (0.0%) |
| **Antenatal care** |  |
| ≥4 visits | 7 (20.6%) |
| 1-3 visits | 23 (67.6%) |
| Not attended | 0 (0.0%) |
| Information missing | 4 (11.8%) |
| **HIV** |  |
| Negative | 22 (64.7%) |
| Positive | 0 (0.0%) |
| Information missing | 12 (35.3%) |
| **Gestational age** |  |
| No information on LMP/gestation weeks | 14 (41.2%) |
| *Of multiparous women:* | *(n=24)* |
| **Previous obstetric history** |  |
| Previous death of child/children* | 10 (41.7%) |
| 1 previous CS | 2 (8.3%) |
| ≥2 previous CSs | 0 (0.0%) |
| *Of all women in the study:* | *(n=34)* |
| **Foetal condition on admission** |  |
| Positive foetal heart rate on admission | 11 (32.4%) |
| *Of women with positive FHR on admission* | *(n=11)* |
| Dexamethasone prophylaxis given | 1 (9.1%) |

* Documentation was insufficient to clearly distinguish perinatal deaths from deaths later in life.

BW, birthweight; g, grams; LMP, last menstrual period

**Table S2** Outcomes for very preterm stillbirths

|  | **Very preterm stillbirths** *BW 1000-1999 g* |
| --- | --- |
|  | N (%) |
| **Mode of delivery** |  |
| *Of all women in the study:* | *(n=34)* |
| Spontaneous vaginal | 27 (79.4%) |
| Vaginal breech | 2 (5.9%) |
| Vacuum extraction | 0 (0.0%) |
| Caesarean section | 4 (11.8%) |
| Mode of delivery unknown | 1 (2.9%) |
| **Maternal outcome** |  |
| *Of all women in the study:* | *(n=34)* |
| Maternal deaths | 1 (2.9%) |
| Post partum haemmorrhage | 3 (8.8%) |
| Episiotomy/spontaneous tears * | 1 (2.9%) |
| *Of vaginal deliveries:* | *(n=27)* |
| Prolonged admission, ≥1 day | 7 (24.1%) |
| *Of caesarean sections:* | *(n=4)* |
| Prolonged admission, ≥5 days | 1 (25.0%) |
| **'Fresh' versus 'macerated' stillbirths** | |
| *Of all women in the study:* | *(n=34)* |
| Fresh | 6 (17.6%) |
| Macerated | 14 (41.2%) |
| Not recorded | 14 (41.2%) |

* Information was insufficient to distinguish between spontaneous vaginal tears and episiotomies.

BW, birthweight; g, grams

**Table S3** Admission and partograph use in case of very preterm stillbirths

|  | **Very preterm stillbirths** *BW 1000-1999 g* |
| --- | --- |
|  | N (%) |
| **Progress on admission and referrals** |  |
| *Of all women in the study:* | *(n=34)* |
| Before labour pain | 2 (5.9%) |
| Latent phase of labour * | 10 (29.4%) |
| First stage of active phase of labour | 10 (29.4%) |
| Second stage of labour | 7 (20.6%) |
| Stage of labour on admission unknown | 5 (14.7%) |
| Referral from smaller health centre | 5 (14.7%) |
| **Partograph use** |  |
| *Of women in first stage, active phase of labour:* | *(n=19)* |
| The partograph at least partially applied | 9 (47.4%) |
| *Of women with the partograph applied:* | *(n=9)* |
| First cervical dilatation in active labour plotted correctly on the alert line | 8 (88.9%) |

* Cervical dilatation <4 centimeter

BW, birthweight; g, grams

**Table S4** Intrapartum surveillance of labour progress and oxytocin use in case of very preterm stillbirths

|  | **Very preterm stillbirths** *BW 1000-1999 g* |
| --- | --- |
|  | N (%) |
| **Surveillance in latent phase of labour** | |
| *Of women admitted before active labour:* | *(n=12)* |
| Assessment of cervical dilatation during active labour | 2 (16.7%) |
| **Assessment of labour progression** | |
| *Of women in first stage of active labour:* | *(n=19)* |
| <5 hrs. between any 2 recordings of cervical dilatation in active labour | 15 (78.9%) |
| <3 hrs. between any 2 recordings of uterine contractions | 15 (78.9%) |
| Alert line crossed | 1 (5.3%) |
| Action line crossed | 0 (0.0%) |

BW, birthweight; g, grams

**Table S5** For very preterm stillbirths, intrapartum surveillance and management of maternal vital signs with focus on severe hypertension and fever.

|  | **Very preterm stillbirths** |
| --- | --- |
|  | *BW 1000-1999 g* |
|  | N (%) |
| **Blood pressure and severe hypertensive disorders** | |
| *Of women reaching active phase of labour:* | *(n=31)* |
| BP recorded at least once during active phase of labour | 25 (80.6%) |
| *Of all women in the study:* | *(n=34)* |
| Severe hypertension (BP ≥160/110) | 12 (35.3%) |
| *Of women with severe hypertension:* | *(n=12)* |
| Analysis for proteinuria recorded | 9 (75.0%) |
| Proteinuria ≥2+ (severe pre-eclampsia)* | 8 (66.7%) |
| Eclampsia / eclamptic fits / convulsions | 0 (0.0%) |
| Antihypertensive treatment initiated | 7 (58.3%) |
| >1 hour between BP recordings | 11 (91.7%) |
| Treatment/observation sheets applied | 5 (41.7%) |
| *Of women with severe pre-eclampsia:* | *(n=9)* |
| Magnesium sulphate treatment initiated | 6 (75.0%) |
| **Temperature and infections** |  |
| *Of all women in the study:* | *(n=34)* |
| Temperature recorded at least once from admission till delivery | 17 (50.0%) |
| Intrapartum fever or infection detected | 0 (0.0%) |

* As information on organ symptoms were limited, cases with severe hypertension and symptoms of organ failure, but no significant proteinuria, were not included here.

BP, blood pressure; BW, birthweight; FHR, foetal heart rate; g, grams
