# Supplementary material for: Stillbirths and quality of care during labour at the low resource referral hospital of Zanzibar: a case-control study
Source: BMC Pregnancy Childbirth. 2016 Nov 10;16:351. doi: 10.1186/s12884-016-1142-2 (PMC5103376; doi:10.1186/s12884-016-1142-2)
Supplement: Additional file 2: — Maternal deaths. Intra-hospital management preceding the three maternal deaths within the study population. (DOCX 39 kb) [file 12884_2016_1142_MOESM2_ESM.docx]

**Additional file 2: Intra-hospital management preceding the three maternal deaths**

**36 years old, G4P3:**

Admitted with fresh vaginal bleeding, severe hypertension, and a history of three previous caesarean sections. The latest of three antenatal care visits was 10 days earlier, where a blood pressure of 213/112 was recorded with insignificant proteinuria. Neither in the antenatal card nor during delivery notes concerning diagnosis and treatment of hypertension was found. Two hours after admission, a stillborn baby was delivered by caesarean section. After an additional two hours, a blood pressure of 80/40 mmHg was measured, but no recordings were made of actions taken. Five hours postoperatively, the woman was found semiconscious and transfusions of blood and Ringers Lactate were ordered. She died after an additional three hours.

**34 years old, G6P5:**

Referred from a neighbouring facility with normal foetal heart rate and cervical dilatation of 4 cm for seven hours (station of head 2/5, weak contractions, membranes intact). The initial plan was to start intravenous Ringers lactate and “review after four hours". Three hours later, an oxytocin infusion of 5 IU in 500 ml Ringers Lactate was started without assessment of foetal and maternal vital signs. No further notes were recorded for an additional two hours before vaginal delivery of a stillborn baby. This was seven hours after crossing the action line. She suffered from retained placenta, which was removed manually. However, she continued bleeding actively, and monitoring and action were inadequate. She died two and a half hours after delivery.

**42 years old, G6P5:**

Admitted in the afternoon in latent phase with meconium-stained liquor and ruptured membranes for three days. A temperature of 39 degrees Celsius was detected on admission and intravenous antibiotics were started. No other maternal vital signs were recorded. Two hours after admission, foetal tachycardia was revealed. No actions were taken, and after an additional 40 minutes the foetal heart rate was no longer detectable. The blood pressure was now assessed, and the woman had severe hypertension (180/80 mmHg). No actions were taken. Two hours later, a fresh stillborn was delivered by vacuum extraction, and at that time the woman died.

G, gravida; P, para
